# PREVALENCE AND BIOPSYCHOSOCIAL PREDICTORS OF POST-STROKE FATIGUE IN PATIENTS WITH MINOR STROKES

**DOI:** 10.2340/jrm.v58.44763

**Published:** 2026-04-21

**Authors:** Anita KJEVERUD, Stein ANDERSSON, Anners LERDAL, Anne-Kristine SCHANKE, Kristin ØSTLIE

**Affiliations:** 1 Department of Physical Medicine and Rehabilitation, Innlandet Hospital Trust, Ottesta; 2 Department of Psychology, University of Oslo, Oslo; 3 Psychosomatic and CL Psychiatry, Division of Mental Health and Addiction, Oslo University Hospital, Oslo; 4 Research Department, Lovisenberg Diaconal Hospital, Oslo; 5 Department of Public Health and Interdisciplinary Health Sciences, Institute of Health and Society, Faculty of Medicine, University of Oslo, Oslo; 6 Research Department, Sunnaas Rehabilitation Hospital, Nesodden, Norway

**Keywords:** fatigue, minor stroke, psychological distress, post-stroke fatigue, physical impairments, cognitive impairments

## Abstract

**Background:**

The majority of stroke patients suffer minor stroke. Little is known about the prevalence of post-stroke fatigue (PSF) and which factors are associated with fatigue in minor stroke patients.

**Objective:**

To investigate the prevalence of PSF in a sample of minor stroke patients and to explore associations between biopsychosocial factors and fatigue 12 months post stroke.

**Methods:**

In this observational study of 72 minor stroke patients fatigue symptoms were measured in the acute phase and 12 months post stroke using the Fatigue Severity Scale (FSS). At 12 months, data on psychological distress symptoms, coping strategies, balance, cognitive function, and self- report of stroke-related symptoms were collected using standardized questionnaires and tests. To explore the possible associations between fatigue and the different variables the Mann–Whitney *U* test and Spearman’s correlations were used. A multiple regression was conducted to identify which of the factors had the strongest association with fatigue

**Results:**

Almost 20% of the sample were fatigue cases 1 year post stroke using a cut off of 5 on the FSS. PSF was strongly associated with having psychological distress symptoms. In univariate analyses, PSF was also associated with fatigue in the acute phase, stroke-related physical impairments, and self-report of symptoms of cognitive impairments.

**Conclusions:**

PSF is present in a subgroup of patients with minor stroke. To identify patients at risk of developing chronic PSF, minor stroke patients should be screened for fatigue early after stroke. A short screening of factors associated with fatigue, such as psychological distress and impaired motor, visual, or cognitive function is also advisable. There is a need for further research in larger samples on PSF and associated factors in minor stroke.

## LAY ABSTRACT

Post-stroke fatigue is a common consequence of stroke and is rated among the worst symptoms to cope with by stroke patients. Little is known about the prevalence of post-stroke fatigue and which factors are associated with fatigue in minor stroke patients, although the majority of stroke patients suffer minor stroke. In this observational study of 72 minor stroke patients, we investigated the prevalence of post-stroke fatigue and explored how biopsychosocial factors such as medical, psychological, physiological, and cognitive factors were associated with fatigue 1 year post stroke. This study indicates that post-stroke fatigue is present in a subgroup of patients with minor stroke. Post-stroke fatigue was associated with psychological distress symptoms, stroke-related impairments, and fatigue in the acute phase. For clinicians, it is important to identify fatigue when present as it impacts on life quality and ability to return to work. Based on our results we suggest a short screening in the early stages after stroke assessing fatigue, psychological distress, stroke-related impairments, and self-report of cognitive and visual dysfunction.

Stroke is one of the leading causes of death and disability worldwide (1). According to a meta study by Alghamdi et al. (2), post-stroke fatigue (PSF) is a severe condition affecting about 48% (95% CI 42–53%) of stroke survivors (2), associated with reduced quality of life, rehabilitation outcome, and ability to work (3–6). Fatigue has been described as a lack of physical and/or mental energy, comprising complex interactions of biological, motor-perceptive, cognitive, emotional, psychosocial, and behavioural factors (7).

Although there is a lack of consensus on the definition of minor stroke (8), current literature suggests that about 60–75% (6, 8–10) of strokes can be classified as minor. Minor stroke patients frequently have few visible disabilities and are often discharged home with little follow-up (11–13). However, research indicates that people with minor strokes also experience lasting impairments such as fatigue (8, 12, 14, 15). A recent study on patients with minor stroke reported fatigue in 53% using a cut-off of ≥ 4 on the Fatigue Severity Scale (FSS) at 3 months post stroke (10). Another study found fatigue in 31% using a cut off of ≥ 5 on the FSS 12 months post stroke (8). Substantial fatigue was found in 65.3% using the Fatigue Assessment Scale in a sample of patients with minor stroke 1 year after stroke onset (6). Nevertheless, a recent review describes a significant knowledge gap on the prevalence of fatigue following minor stroke (14).

Sequalae such as subtle motoric dysfunction, cognitive, or visual impairments, may be associated with PSF in minor stroke (4, 16, 17). A recent cross-sectional study of patients with predominantly minor stroke found impairments in either cognitive function or motor function in about one-third of the patients (9). Cognitive sequalae such as reduced information-processing speed, attention, visual memory, or executive dysfunction may be more subtle in patients with minor compared with major stroke and may therefore go unnoticed (18). However, they can still impact on functional recovery, quality of life, and return to work (19).

It has also been found that the ability to work following minor stroke is negatively affected by depression or fatigue (20, 21). Fatigue and depression are often concomitant (22), and depression may also be a consequence of fatigue if ability to return to work and other aspects of life are hampered by PSF.

In this exploratory study we have 2 aims:

- First, we aim to investigate the prevalence of PSF in a sample of stroke patients suffering minor stroke in the acute phase and at 1e year post stroke.
- Second, we aim to explore how biopsychosocial factors such as severity of physical disability, cognitive function, pain, sleep quality, coping strategies, and psychological distress are associated with fatigue in patients with minor stroke at 12 months post stroke.

## MATERIALS AND METHODS

### Study sample and procedures

We performed an observational study recruiting stroke patients from 1 hospital in Innlandet Hospital Trust, Norway, between February 2017 and October 2019.

Patients were eligible for the study if they were 18 years or older and had ischaemic stroke according to the International Classification of Disease 10 (ICD-10 I60-I63.9, I64) confirmed by trained physicians in the acute hospital. Exclusion criteria for participation were intracerebral haemorrhagic stroke, other somatic or psychiatric debilitating diseases, or severe language or cognitive dysfunctions causing potential problems with data collection. Patients were included if the National Institutes of Health Stroke Scale (NIHSS) score was < 5 at discharge, indicating a minor stroke and few stroke-related impairments. 28% of the original sample (*n* = 78) had thrombolytic treatment, in some cases leading to a significant drop in NHISS score from admission to discharge. The NIHSS score on discharge from hospital was thus chosen as this reflected their perceived level of impairment upon return to everyday life or rehabilitation.

**Fig. 1:**
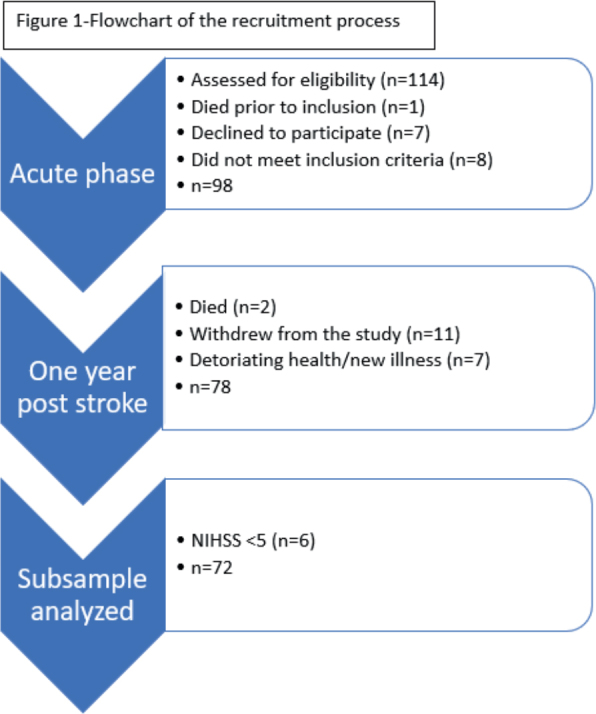
Flowchart of the recruitment process.

A total of 72 patients participated in the data collection in the acute phase, defined as within the first 2 weeks after stroke onset, and 12 months post stroke. The recruitment and inclusion process is described in Fig. 1.

The patients met at the clinic 12 months post-stroke. Data were collected using standardized questionnaires, neuropsychological assessment of cognitive function, and physical tests, as detailed in Table SI. Furthermore, data from the acute phase were collected from the patient’s medical records and included stroke classification, NIHSS score, comorbidities, demographic, and medical variables. Data on fatigue severity were collected at both time-points through administration of the Fatigue Severity questionnaire-7 (FSS-7). Data on pre-stroke fatigue, cohabitation, work status, and possible new comorbidities were obtained through a short interview, and the BMI was calculated at the clinic. The main outcome measures are also detailed below.

### Outcome measures

*Fatigue.* Fatigue PSF was assessed using the Fatigue Severity Scale (FSS). In accordance with English et al. (23), the 7-item version of the instrument was chosen. The FSS is the most frequently used self-report instrument for measuring fatigue in stroke populations (24). The scale includes items such as “Fatigue is among my three most disabling symptoms” and “Fatigue interferes with carrying out certain duties and responsibilities”. Responses reflect the degree to which one agrees with each statement on a Likert scale of 1–7 based on experience within the last 7 days (25). A mean score is calculated, with higher scores indicating more severe fatigue. The FSS has shown acceptable validity and reliability across different clinical populations (26).

In accordance with Lerdal et al. (27) we used a cut-off of 5 for identifying clinically significant fatigue (17, 28). For lack of a consensus on a clinically meaningful cut-off to identify fatigue cases (29), we used the mean FSS-7 scores as a continuous variable when exploring associations between fatigue severity and predictor variables.

*Demographic and medical variables.* We collected information on age, employment status, and educational level from medical records and by personal interview.

*National Institute of Health Stroke Scale (NIHSS)* is an 11-item scale used to objectively quantify stroke impairment. It is scored during admission to hospital in the acute phase and on discharge. The scores range from 0–42; higher scores equal more impairment. Scores 0–4 indicate minor stroke, scores 5–15 a moderate stroke, scores 16–20 moderate to severe stroke, and 21–42 severe stroke (30). In this study, in accordance with Lyden et al. (30, 31), minor stroke is defined as a NIHSS score of < 5.

*Questionnaires and methods to assess physical and cognitive function.* The Barthel Index for ADL (activities of daily living) and Berg test of balance were used to measure level of disability and physical function. Subtests from the Delis–Kaplan Executive Function System, the WAIS IV, and the Halstead–Reitan neuropsychological test battery were used to assess cognitive function. Standardized questionnaires were used to collect data on psychological distress, pain, sleep quality, and symptoms of cognitive impairments. For details on questionnaires and methods to assess physical and cognitive function, see Table SI.

### Data analyses

Statistical analyses were performed using SPSS software version 23 (IBM Corp, Armonk, NY, USA).

The Mann–Whitney *U* test was used to analyse how categorical variables such as pre-stroke fatigue or cohabitation were related to fatigue severity. Spearman’s correlations were conducted to explore the relationships between scores on the FSS-7 and the continuous variables. Spearman was chosen because the scores on the FSS-7 at 12 months post stroke were not normally distributed.

To reduce the risk of Type I error due to multiple testing, we set the level for statistically significant differences to *p* ≤ 0.01. Associations between fatigue scores and predictor variables significant at the 0.05 level are also reported but should be regarded as tendencies.

A multiple regression was conducted to identify which of the factors had the strongest association with fatigue. The screening criterion for the multivariate analyses was set to *p* ≤ 0.05 to avoid excluding clinically and theoretically important variables. The categorical variable “cohabitation” was transformed into a dummy variable before analyses.

Values from a Durbin–Watson analysis were acceptable within the range of 1.5 to 2.5 when controlling for possible autocorrelations.

### Ethics

Before inclusion, all participants signed informed consent. The study was approved by the Regional Ethics Committee for Medical and Health Research Ethics in South-Eastern Norway (Ref. 2016/589/REK Sør-Øst C).

## RESULTS

### Demographic and medical characteristics

Of the 72 patients included in the analyses, a slight majority were male and the mean age on admission was 66.4 (SD 9.7). One year post stroke, the most common comorbidities were hypertension and diabetes mellitus; no association was found between comorbidities and fatigue. However, those who reported higher fatigue severity tended to have a higher NIHSS score on discharge from the acute hospital (*r*^s^.28; *p* = 0.02) (for details on demographic and medical characteristics, see Table I). The majority of the patients were living with a partner 1 year post stroke; living alone was associated with fatigue; z = (–2.13); *p* = 0.03. None of the other demographic or medical factors were associated with fatigue severity.

**Table I:** Demographic, clinical, physical, and psychological characteristics of the sample *(n*=72)

Variable	
Demographic	
Age on admission (years), mean (SD^1^)	66.4 (9.7)
Sex (male/female)	48 / 24
Cohabitation (partner/alone)	57 / 15
Working at stroke onset (yes/no)	34 / 38
Clinical	
NIHSS^2^ on admission, mean (SD)	2.8 (1.3)
NIHSS on discharge, mean (SD)	0.8 (1.4)
Thrombolysis, *n* (%)	20 (27.8%)
BMI^3^, mean (SD)	26.6 (4.3)
Comorbidities, mean (SD)	1.8 (1.4)
Pre-stroke fatigue (yes), *n* (%)	17 (23.6%)
Physical	
Barthel Index (ADL^4^), mean (SD)	19.6 (1.8)
Berg Balance Test, mean (SD)	53.2 (8.1)
Cognitive	
Visuomotor function, mean (SD)	47.2 (8.5)
Processing speed, mean (SD)	47.9 (8.4)
Attention, mean (SD)	49.8 (10.4)
Executive function, mean (SD)	8.1 (8.5)
Self-report	
Self-reported stroke-related symptoms (RPQ^5^), mean (SD)	13.7 (10.3)
Psychological distress (HSCL- 25^6^), mean (SD)	1.3 (0.3)
Avoidant coping	16.3 (3.5)
Approach coping	26.0 (6.0)
Sleep quality (PSQI^7^), mean (SD)	4.2 (3.4)
Pain (Numeric Rating Scale), mean (SD)	2.0 (2.4)

1 Standard deviation,

2 National Institute of Health Stroke Scale,

3 Body Mass Index,

4 Activities of Daily Living,

5 Rivermead Post Concussion Symptoms Questionnaire,

6 Hopkins Symptom Checklist-25,

7 Pittsburgh Sleep Quality Index

### Fatigue symptoms, fatigue-caseness, and pre-stroke fatigue

At one year post stroke, about a fifth of the patients were fatigue cases based on a cut off of 5 at the FSS-7; of these patients, about 40% reported pre-stroke fatigue as well. Patients who reported pre-stroke fatigue also reported more fatigue 1 year post stroke; however, the association was not significant (*p* = 0.06). In general, the patient’s FSS scores were stable through the first year post stroke, although there were individual differences. More severe fatigue in the acute phase predicted fatigue 1 year post stroke (*r*^s^ = 41; *p* < 0.001) (for details see Table II).

**Table II:** Fatigue in the acute phase and at 12 months post stroke (*n* = 72)

Item	Acute phase	12 months post stroke
FSS scores (mean, SD)	3.6 (1.5)	3.3 (1.5)
FSS scores (median, IQR)	3.6 (2.7)	3.2 (2.4)
No. of fatigue cases (FSS ≥ 5), *n* (%)	15 (20.8)	14 (19.4)
No. of fatigue cases with pre-stroke fatigue, *n* (%)	9 (60%)	6 (42%)

FSS: Fatigue Severity Scale; SD: standard deviaiton; IQR: interquartile range.

**Table III:** Univariate analyses of demographic, clinical, physical, and psychological characteristics factors and FSS scores 1 year post stroke (*n* = 72)

Variable	Spearman’s Rho /z (*r*^1^)	*p*-value^2^
Demographic		
Age on admission (years), Spearman’s Rho	–0.01	0.91
Sex (male/female), z (*r*)	–1.25 (–0.15)	0.21
Cohabitation, z (*r*)	–2.13 (–0.25)	0.03*
Working at stroke onset (yes/no), z (*r*)	–2.00 (–0.24)	0.84
Clinical		
NIHSS^3^ on admission, Spearman’s Rho	0.016	0.17
NIHSS^3^ on discharge, Spearman’s Rho	0.28	0.02*
Thrombolysis, *n* (%), z (r)	–0.03 (–0.00)	0.98
BMI^4^, Spearman’s Rho	–0.04	0.78
Comorbidities, Spearman’s Rho	0.20	0.10
Pre-stroke fatigue (yes), z (*r*)	–1.9 (–0.22)	0.06
Physical		
Barthel Index (ADL^5^), Spearman’s Rho	–0.19	0.11
Berg Balance Test, Spearman’s Rho	–0.48	< 0.001**
Cognitive		
Visuomotor function, Spearman’s Rho	–0.02	0.84
Processing speed, Spearman’s Rho	0.06	0.63
Attention, Spearman’s Rho	0.10	0.40
Executive function, Spearman’s Rho	0.04	0.73
Self-report		
** **FSS-scores in the acute phase, Spearman’s Rho	0.41	< 0.001**
* *Self-reported stroke-related symptoms (RPQ^6^), Spearman’s Rho	0.42	< 0.001**
Psychological distress (HSCL-25^7^), Spearman’s Rho	0.52	< 0.001**
Avoidant coping, Spearman’s Rho	0.37	0.01*
Approach coping, Spearman’s Rho	0.33	0.01*
Sleep quality (PSQI^8^), Spearman’s Rho	0.25	0.03*
Pain (Numeric Rating, Spearman’s Rho	0.16	0.17

1 Magnitude of the difference between the 2 independent groups (*r*),

2 significance level,

3 National Institute of Health Stroke Scale,

4 Body Mass index,

5 Activities of Daily Living,

6 Rivermead Post Concussion Scale,

7 Hopkins Symptom Checklist-25,

8 Pittsburgh Sleep Quality Index.

### Associations between self-reported fatigue and clinical characteristics in the sample; physical and cognitive characteristics and self-report of symptoms

At 1 year post stroke, most of the patients in the sample were independent in activities of daily living. However, having somewhat impaired balance was significantly associated with fatigue symptoms (*r*^2^ = –0.48; *p* < 0.001).

There was no association between the scores on cognitive measures and fatigue severity. There was, however, a strong association between reporting more stroke-related cognitive and sensory symptoms on the RPQ (*r* = 0.50, *p* < 0.001) and fatigue severity. On the RPQ, the following items had the strongest correlation with fatigue symptoms: feelings of dizziness (*r*^s^ = 0.38; *p* < 0.001), forgetfulness, poor memory (*r*^s^ = 0.51, *p* < 0.001), and taking longer to think (*r*^s^ = 0.39, *p* < 0.001). There was also an association between fatigue symptoms and poor concentration (*r*^s^ = 0.35, *p* = 0.00, noise sensitivity (*r*^s^ = 0.31, *p* = 0.01) and blurred vision (*r*^s^ = 0.30, *p* = 0.01).

Patients who reported more psychological distress experienced more fatigue than those with fewer symptoms of psychological distress (*r*^s^ = 0.52, *p* < 0.001). Severe fatigue symptoms were associated with using more avoidant coping (*r*^s^ = 37; *p* = 0.002) and also associated with using more approach-coping strategies (*r*^s^ = 0.33; *p* = 0.008).

Those with poorer sleep quality tended to experience more fatigue (*r*^s^ = 25; *p* = 0.03). Most patients in this study did not report severe pain, and no association between pain and fatigue was found.

A multivariate analysis that included variables that were associated with the scores on the FSS at 12 months post stroke with a *p*-value of ≤ 0.05 was conducted, using linear regression. The following variables were entered into the model; FSS scores in the acute phase, NHISS score on discharge, cohabitation, Berg test of balance, psychological distress symptoms, avoidant coping, approach coping, and sleep quality. The present model suggests that 33% of the variance in fatigue could be attributed to having psychological distress symptoms. For details, see Table IV).

**Table IV:** Multivariate regression analysis of factors associated with FSS at 12 months post stroke

Predictor	B^1^	95% CI	β^2^	*p*-value^3^
Cohabitation	1.39	–89–3.87	0.14	0.23
NIHSS on discharge	0.18	–12–0.47	0.15	0.23
Berg Balance Test	–0.01	–0.06–0.03	–0.08	0.53
FSS scores in the acute phase	0.17	–0.12–0.46	0.16	0.46
* *Self-reported stroke-related symptoms (RPQ4^4^)	0.00	–0.04–04	0.03	0.85
Psychological distress (HSCL-25^5^)	1.34	0.02–2.68	0.31	0.04*
Avoidant coping	0.03	–0.10–0.16	0.06	0.69
Approach coping	0.02	0.09–67	0.09	0.50
Sleep quality (PSQI^6^)	0.02	–0.09–0.14	0.05	0.68
Cohabitation	1.39	–89–3.87	0.14	0.23

1 Unstardardized coefficient,

2 Standardized coefficient,

3 Significance level. The following variables were used in the analyses based on a *p*-value of <=0.05: FSS score in the acute phase, NHISS on discharge, Berg Balance Test, Self Reported Stroke Related Symptoms,

4 Rivermead Post Concussion Symptoms questionnaire, Psychological distress,

5 Hopkins Symptom Checklist-25, Avoidant coping, Approach coping, Sleep quality,

6 Pittsburgh sleep Quality Index), Cohabitation.

## DISCUSSION

### Prevalence of PSF

In this study, we explored the prevalence of PSF and associates of fatigue in patients with minor stroke. We found fewer fatigue cases 1 year post stroke (19.4%) in our study than has been found in other studies, such as in the studies by Vitturi et al. (10), Morsund et al. (8) or Einstad et al. (9), who reported fatigue in 53%, 31%, and 65% in their samples. This variance may be due to differences in how many patients received thrombolytic treatment in the different studies; thrombotic treatment led to a significant reduction in stroke-related impairments in our sample. Furthermore, there is a challenge in the comparison of data on prevalence of PSF across studies, due to lack of consensus on methodology used for identifying substantial fatigue. While Vitturi et al. used a cut-off of 4 on the FSS, we used a cut off of 5 in accordance with Lerdal et al. (32). If we had used a cut-off of 4, 29.4% would have been fatigue cases 12 months post stroke, making the results more similar regarding prevalence. However, using a cut-off of 4 to identify cases may lead to an overestimation of severe fatigue as the mean score is reported to be 3.67 (SD 1.17) in the general Norwegian population (27).

Different definitions of minor stroke present another challenge in comparing results across studies. (14). Some use a NIHSS score of < 4 (10) while others use a NHISS score of <5, yet another an NIHSS score = < 5 (30, 31). Some studies use a score of ≤ 2 on the modified Rankin Scale (mRS) to define minor stroke (6, 10). We chose a cut-off of < 5 on the NIHSS in accordance with Lyden et al. (33). A similar approach is also used in comparable studies (9, 34).

A majority of stroke survivors experience minor stroke (10, 11). A prevalence of PSF of 20% in minor stroke survivors thus accounts for a large number of people.

### Association between PSF and biopsychosocial factors

We found that experiencing fatigue post stroke was associated with both physical and psychological factors, and with self-reported cognitive impairment.

The strong association between symptoms of psychological distress and fatigue severity is in line with previous studies showing an association between psychological distress and PSF (35–37). For some patients, depression might be a risk or maintaining factor for fatigue, or a consequence of fatigue impacting on the capacity for participation in various aspects of life. Further, in univariate analyses there was also a strong association between living alone and experiencing fatigue. Living alone may imply less support from significant others, and thereby possibly lead to more psychological distress and fatigue.

The association of impaired balance being associated with fatigue is in line with other studies reporting that severity of PSF is associated with severity of disability (5) and functional decline (26). PSF can in some patients be attributed to the increased effort associated with the use of new movement patterns and increased effort in coping with impairments in everyday life (38). It is likely that a stronger association between fatigue and severity of disability would have been found in a sample that included patients with moderate to severe stroke, i.e., in a sample of patients with more residual impairments because of their stroke.

Scores within the normal range on cognitive tests in the majority of the patients in this sample are to be expected; however, the finding that self-report of symptoms of cognitive impairments such as using longer time to think, difficulty concentrating, and memory dysfunction was associated with experiencing more fatigue symptoms is a highly relevant finding for clinical practice. This indicates the importance of being aware of these more subtle signs of cognitive impairment to identify fatigue, even though the patients' neuropsychological tests are inconspicuous. Small deviations from population norms might represent a within-person decline. Unfortunately, we do not have any measures of the participants’ cognitive function before the stroke. The subjective cognitive complaint may be an expression of how they experience cognitive decline after their stroke. Further, subtle cognitive impairments contribute to fatigue, especially in situations that demand sustained cognitive effort. It may also be that these symptoms overlap with mental fatigue, and that mental fatigability is expressed as difficulties with concentration and forgetfulness*.*

Inspecting which items on the RPQ showed the highest correlation with fatigue, we found a tendency for self-reported visual problems to be associated with PSF. This is in line with the study by Pedersen et al. (17). Slow visual tracking and impaired fine-motor control may make both mental and physical tasks more effortful and thus cause fatigue, and visual rehabilitation may reduce fatigue in some stroke survivors.

Most likely is that the lack of strong association between pain or having comorbid conditions and fatigue in our study is due to a low frequency of these symptoms in our sample of minor stroke, compared with the stroke population in general.

Finally, several studies have found a strong association between pre-stroke fatigue and post-stroke fatigue (32, 39). In this study, although we found that those who reported pre-stroke fatigue tended to have higher scores on the FSS 1 year post stroke, the association was not significant. Fatigue might thus be a new experience for some minor stroke patients; in our sample 58% of the fatigue cases did not report pre-stroke fatigue. There was, however, a strong association between fatigue in the acute phase and fatigue 1 year post stroke. Thus, in line with previous studies, early fatigue predicts more long-lasting fatigue (39, 40). This implies that, in many cases, long-lasting fatigue may be predicted from experiencing fatigue symptoms shortly after a stroke. Thus, early assessment of fatigue severity is warranted.

### Implications for clinical practice and further research

The results in this study support earlier findings that some patients suffer from PSF after minor stroke. Clinically, it is crucial to identify those who are vulnerable. Fatigue in the early phase post stroke predicts fatigue in later stages post stroke. Thus, an early screening of fatigue and associated factors such as stroke-related impairments is advisable to prevent the development of long-lasting, or even chronic, PSF. Primary healthcare workers may be a resource in both identifying possible unmet needs for follow-up, and in the management of the symptoms.

To date, there is relatively sparse research on treatment strategies aimed at alleviating PSF (41). Graded exercise combined with cognitive therapy (42) or psychoeducation (43) has been suggested.

Perceiving the future as manageable and using adaptive coping strategies has been suggested as a protective buffer against fatigue (44, 45). Hence, coping strategies and self-efficacy beliefs may also be possible targets for intervention. It may be that we had too few participants to capture a clear association between the use of different coping styles and fatigue, or that the unclarity results from response bias such as generally agreeing with the statements on the scale used. The possible associations between coping styles, self-efficacy beliefs, and PSF should be studied further in future research with larger samples. Qualitative research and semi-structured interviews may expand our understanding of the reasons for fatigue, and as such contribute to choices for instruments and hypotheses in future research.

In further research, a focus should also be on achieving consensus on how to measure fatigue and how to define minor stroke, yielding comparable data from different samples. Clinically, definitions of minor stroke might impact treatment decisions (46).

### Strengths and weaknesses

A key strength of this study is having a multidimensional protocol consisting of demographic, medical, physical, cognitive, and psychological variables. Assessment of fatigue in the acute phase is another strength, including specific measures of coping strategies and cognitive function.

The study also has limitations. The sample is small, limiting the statistical strength of the analyses. The group of fatigue cases is small, further limiting the statistical power, and statistical outliers might affect the result. The sample being small, the findings from this exploratory study must be regarded as hypothesis-generating. There is a need for further research in larger samples, preferably multicentre studies. Further studies may include more measures in the acute phase to investigate which factors may predict long-lasting fatigue. For instance, if depression in the acute phase predicts fatigue 1 year post stroke, an early assessment and treatment of symptoms of depression and/or psychological distress is warranted.

In general, when conducting research on patients with minor stroke, definitions of minor stroke might lead to potential misclassification of the study population. It has been found that patients with left hemisphere strokes have higher NIHSS scores than patients with right hemisphere strokes due to the weight on language deficits (aphasia) in the scoring system (46).

Associations between fatigue and predictor variables at a significance level of *p* < 0.05 are described. However, there is a risk of type I error due to multiple testing and these findings should be regarded as tendencies. In order to minimize this risk, we reduced the level of significance from *p* < 0.05 to *p* < 0.01.

### Conclusions

This exploratory study that measures fatigue in the acute phase and at 1 year post stroke indicates that PSF is present in a subgroup of patients with minor stroke. To identify patients at risk of developing chronic PSF, minor stroke patients should be screened for fatigue early after stroke. A short screening of factors associated with fatigue, such as psychological distress, and impaired motor, visual, or cognitive function, is also advisable.

It is important to identify fatigue when present as it impacts on the ability to return to work, on capacity for those who do return to work, and on everyday life and quality of life (21). Given the diversity of the factors associated with PSF, fatigue assessment and interventions should be multidisciplinary. Further research on larger samples is needed for in-depth exploration of how different biopsychosocial factors interact and impact on the development and severity of post-stroke fatigue following minor stroke.

## Supplementary Material


