# Supplementary material for: PREVALENCE AND BIOPSYCHOSOCIAL PREDICTORS OF POST-STROKE FATIGUE IN PATIENTS WITH MINOR STROKES
Source: J Rehabil Med. 2026 Apr 21;58:44763. doi: 10.2340/jrm.v58.44763 (PMC13107390; doi:10.2340/jrm.v58.44763)
Supplement: Supplementary file 1 [file JRM-58-44763-s1.pdf]

Supplementary material has been published as submitted. It has not been copyedited, or typeset by Journal of Rehabilitation Medicine

Table SI Summary of questionnaires, physical and cognitive measures used at 12 months post-stroke

	Description	Classification	References
**Physical function and level of disability**			
Barthel Index for ADL	10 item scale that assesses level of disability in activities of daily living	Range of scores: 0-20<br>Higher scores=higher level of independence	(47)
Berg test of balance	14 step test that assesses balance and risk of falling	Range of scores: 0-56<br>Higher scores= better balance<br>Scores <45 indicates risk of falling	(48)
Body mass index (BMI)	BMI was calculated as the weight in kilograms divided by the square in meters.		
The comorbidity questionnaire	consists of 18 items covering different medical conditions (49)	A sum score was made of the total of comorbidities. Medical records were also inspected	
Pre-stroke fatigue	was assessed according to Lerdal (32) through questions in the short interview at12 months post-stroke. Fatigue was defined as substantial if it had lasted for at least 3 months before the stroke.	Fatigue was defined as substantial if it had lasted for at least 3 months before the stroke and affected the ability to perform daily activities.	
**Cognitive domains[1]**			
Visuo-motor speed	Grooved pegboard test: asses fine-motor speed and control<br>Condition 1 and 5 from Trail Making Test (TMT). Condition 1: baseline visual scanning speed and condition 5: fine-motor speed.	Higher score= better function	(50, 51)
Processing speed	Condition 2 and 3 from the TMT<br>Conditions Color naming and Word reading from Color-Word Interference test (CWIT).	Higher score=better function	(52)
Attention	Digit span from WAIS IV: short term memory and working memory	Higher scores=better function	(53)

Executive function	Condition 4 from the TMT: cognitive set shifting<br>Condition 3 and 4 from the CWIT: Inhibition and inhibition/switching	Higher scores =better function	(51)
**Questionnaires**			
Rivermead Post Concussion Symptoms Questionnaire (RPQ).	16 items assessing self-reported cognitive function, vision, fatigue and physical function. Rate of symptom severity in the last 24 hours on an ordinal scale ranging from 0 (no problems) to 4 (a severe problem).	Total scores range 0-64, higher scores= worse symptom severity.	(54).
Hopkins Symptom Checklist 25 (HSCL-25)	A 25 item questionnaire measuring symptoms of psychological distress, including symptoms of anxiety and depression	Higher mean scores indicate higher degree of depression and/or anxiety	(55)
BRIEF-Cope[2]	A 28 item questionnaire that assesses coping-strategies to deal with demands in life. Avoidant and approach coping strategies are operationalized in accordance with Eisenberg[1]	Higher scores =more use of either avoidant or approach strategies.	(56, 57)
Pittsburg sleep quality index (PSQI)	A 19 item questionnaire that assesses sleep quality over a one-month time-period.	Higher scores= worse sleep quality<br>Score of 5 or higher is associated with sleep-disturbances	(58)
Pain severity	Assessed using a Numeric Rating Scale from 0 (no pain)-10 (worst pain possible) over the last week	Higher scores= more pain	(59)

[1]Neuropsychological assessment included tests covering cognitive domains of visuo-motor speed, processing speed and attention/executive function (see table 1). Test scores were converted to T-scores. A T-score of 50 (SD =10) represents the normative mean. A T-score of 35 (< 1.5 SD from normative mean) is defined in the clinical impaired range.

[2] Avoidant coping: comprised of the scores on the subscales of denial, substance use, venting, behavioral disengagement, self-distraction and self-blame (range of scores 12-48). Approach coping: comprised of the sub-scales of active coping, positive reframing, planning, acceptance, seeking emotional support, seeking informational support (range of scores: 12-48)
